# Blood pressure‐independent inhibition of Marfan aortic root widening by the angiotensin II receptor blocker valsartan

**DOI:** 10.14814/phy2.14877

**Published:** 2021-05-27

**Authors:** Arash Y. Tehrani, Zoe White, Nadia Milad, Mitra Esfandiarei, Michael A. Seidman, Pascal Bernatchez

**Affiliations:** ^1^ Centre for Heart Lung Innovation University of British Columbia Vancouver BC Canada; ^2^ Department of Anesthesiology Pharmacology & Therapeutics University of British Columbia Vancouver BC Canada; ^3^ Department of Biomedical Sciences College of Graduate Studies Midwestern University Glendale Arizona USA; ^4^ Centre for Heart Lung Innovation Department of Pathology and Laboratory Medicine University of British Columbia Vancouver BC Canada

**Keywords:** endothelium, Marfan syndrome, nitric oxide

## Abstract

Marfan syndrome (MFS) is a genetic disorder that results in accelerated aortic root widening and aneurysm. However, management of MFS patients with blood pressure (BP)‐lowering medications, such as angiotensin II (AngII) receptor blocker (ARB) losartan, continues to pose challenges due to their questionable efficacy at attenuating the rate of aortic root widening in patients. Herein we investigate the anti‐aortic root widening effects of a sub‐BP‐lowering dose valsartan, an ARB previously linked to non‐BP lowering anti‐remodeling effects. Despite absence of BP‐lowering effects, valsartan attenuated MFS aortic root widening by 75.9%, which was similar to a hypotensive dose of losartan (79.4%) when assessed by ultrasound echocardiography. Medial thickening, elastic fiber fragmentation, and phospho‐ERK signaling were also inhibited to a similar degree with both treatments. Valsartan and losartan decreased vascular contractility ex vivo between 60% and 80%, in a nitric oxide (NO)‐sensitive fashion. Valsartan increased acetylcholine (Ach)‐induced vessel relaxation and phospho‐eNOS levels in the aortic vessel supporting BP‐independent activation of protective endothelial function, which is critical to ARB‐mediated aortic root stability. This study supports the concept of achieving aortic root stability with valsartan in absence of BP‐lowering effects, which may help address efficacy and compliance issues with losartan‐based MFS patient management.

## Graphical abstract

Inhibition of Marfan aortic root remodeling and widening with blood pressure lowering medications losartan and valsartan. However, valsartan was used at sub‐blood pressure lowering dose, therefore implying blood pressure independence.

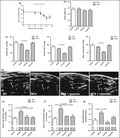

## 1 INTRODUCTION

Marfan syndrome (MFS) is an autosomal dominant disorder caused by mutations in the *FBN1* gene, which codes for fibrillin‐1, a major component of microfibrils and elastic fibers (Dietz et al., 1991). Fibrillin‐1 mutations lead to a range of elastic tissue abnormalities that include elastic fiber fragmentation and disorganization; in the cardiovascular system, this results in aortic dilation, particularly in the aortic root, which contributes to an early incidence of dissections, aneurysm and mortality due to rupture if unmanaged (Cañadas et al., 2010). At the molecular level, loss of fibrillin‐1 integrity can result in heightened transforming growth factor beta (TGF‐β) signaling, resulting in pathological aortic remodeling although how this occurs is a subject of controversy (Franken et al., 2015; Mallat et al., 2017; Park et al., 2019; Wei et al., 2017).

Another source of debate is the pharmacological management of MFS‐associated aortic root widening. Heart‐specific β‐adrenoreceptor blockers have traditionally been the blood pressure (BP)‐lowering drug of choice to reduce the rate of thoracic aortic aneurysms and by extension MFS aortic root widening despite mild therapeutic evidence (Koo et al., 2017). Treatment with losartan, an angiotensin II (AngII) receptor type 1 (ATR1) blocker (ARB) with unique anti‐TGF‐β effects, resulted in decreased rate of aortic root dilation in a rodent model of MFS and in a small scale trial in MFS patients refractory to atenolol (Brooke et al., 2008; Habashi, 2006). However, large‐scale trials failed to document the expected superiority of losartan over atenolol at reducing aortic root dilation rates (Forteza et al., 2016; Lacro et al., 2014), drawing attention to the complex mechanisms that govern aortic root remodeling in MFS.

Recently, we have shown that hypotensive MFS mice with blunted ATR1 expression develop unabated aortic root widening while remaining fully responsive to losartan (Sellers et al., 2018). This suggested that lowering of BP or inhibition of AngII signaling might be of low therapeutic value in MFS, lending credence to the poor efficacy of angiotensin converting enzyme inhibitors (ACEi) against MFS aortic widening (Singh & Lacro, 2016). Instead, our work in the well‐established MFS mouse model suggests that losartan reduces aortic root widening by activating protective endothelial function and nitric oxide (NO) bioavailability in the aorta (Sellers et al., 2018), as hinted by others (Watanabe et al., 2005), which could correct chronic MFS‐associated endothelial abnormalities (Chung et al., 2007; Jiménez‐Altayó et al., 2018; Oller et al., 2017; Syyong et al., 2009; Wilson et al., 1999; Yang et al., 2010). Interestingly, the ARB valsartan has been shown to prevent abdominal aortic aneurysms (AAA), which are notoriously refractory to pharmacotherapy, independently of its BP lowering effect. Hence, although losartan has been used in prior studies at a BP lowering dose, we chose to compare these results with sub‐BP lowering doses of valsartan in MFS mice (Sellers et al., 2018). We show that low‐dose valsartan is as effective as losartan at reducing aortic root widening and increasing endothelial function despite profound differences in their BP‐lowering effects. Our data suggest that low‐dose valsartan could improve clinical outcomes and minimize common side effects associated with BP lowering medications, while increasing long‐term compliance and ameliorating quality of life in MFS patients.

## 2 MATERIALS AND METHODS

### 2.1 Animals

MFS mice (*FBN1 C1039G* ^+/−^) were originally supplied from the laboratory of Dr. Harry Dietz (Johns Hopkins School of Medicine) and back‐crossed to wild‐type C57BL/6J mice (Jackson laboratory stock 00664) for at least three generations. All animals were housed on a standard 12‐h light/dark cycle, in a temperature‐regulated facility, fed a regular chow diet (LabDiet 5001), and all experiments approved by the UBC Animal Care Committee. Mice were sacrificed at 24 weeks of age under inhaled terminal anesthesia (3.5% v/v isoflurane at 1.5 L O_2_) followed by cervical dislocation.

### 2.2 Drug treatment

Six‐week‐old mice were randomly assigned to receive clinical formulations of losartan, valsartan or vehicle (drinking water) until time of sacrifice at 24 weeks of age. Losartan was administered at a previously established sub‐maximal dose (0.6 g/L in drinking water) in terms of BP lowering and aortic root stability ((Koo et al., 2017) and unpublished observations, both with 1.2 g/L), and we established a maximal non‐BP lowering dose of 30 mg/kg for valsartan (Habashi, 2006; Sellers et al., 2018; Yang et al., 2009). Drugs in drinking water were replaced three times per week, and dosages were continuously adjusted based on changes in body weight and volume of water consumed per cage (averaged per day).

### 2.3 Non‐invasive BP measurements and high‐resolution ultrasound imaging

Systemic BP was non‐invasively measured by a tail cuff system (CODA 2, Kent Scientific) as previously published (Sellers et al., 2018). Mice were anesthetized (1.5–2% v/v isoflurane at 1.5 L O_2_), the tail inserted into an inflatable cuff and, following a 10‐min acclimation period, systolic and diastolic BP were measured over 15 cycles and averaged over the last 10 cycle measurements. For echocardiograms, mice were anesthetized and imaged using a VisualSonics Vevo 2100 system with a MS‐550D 40‐MHz probe by a technician blinded to genotype and treatment group. Ascending aorta and aortic root measurements at the level of the Sinus of Valsalva were averaged from multiple measurements taken in both M and B mode as previously described (Lee et al., 2016).

### 2.4 Measurements of isometric force

After euthanasia, aortic rings (2 mm) were cut from each mouse aorta and mounted in a small vessel myograph (AS Danish Myotechnology), stretched to the optimal tension (6.0 mN) for 30 min and challenged with 30 mmol/L KCl, phenylephrine (PE) and acetylcholine (3 nM–100 μM) without or with NO blocker N^ω^‐nitro‐l‐arginine methyl ester (L‐NAME, 200 mM) as previously described (Chung et al., 2007).

### 2.5 Histology

Histology was performed on hearts fixed in 10% buffered formalin. 5 μm cross‐sections were cut throughout the aortic root at the Sinus of Valsalva as previously described (Sellers et al., 2018). Aortic medial thickening and average elastic fiber length (fragmentation) were assessed at the Sinus of Valsalva on slides stained with Verhoeff‐van Geison staining as previously described (Cui et al., 2014). For immunohistochemistry, sections were deparaffinized and rehydrated, followed by antigen retrieval (10 mM citrate buffer, pH 6) and quenching of endogenous peroxidase with 3% hydrogen peroxide in methanol for 15 min at room temperature. Sections were blocked for 2 h with 10% normal serum plus 1% BSA in TBS and probed with rabbit anti‐phospho‐p44/42 MAPK (ERK 1/2; 1/200; Cell Signaling Technology, Cat# 9101S) and rabbit anti‐p‐eNOS (phospho‐eNOS‐Ser1177; 1:100; Invitrogen, Cat# PA517917). Immunoreactivities were detected based on secondary biotinylated antibodies, followed by visualization with DAB (Vector Laboratories), sections were counterstained with hematoxylin if necessary, and images were acquired using an Aperio ScanScope AT2 scanner.

### 2.6 Statistical analysis

GraphPad Prism software 6.01 was used for all analyses. Values are expressed as mean ± standard error of the mean (SEM) with a *p* < 0.05 value considered significant, *n* = 5–6 for all groups. One‐way analysis of variance (ANOVA) was used to compare multiple groups with Tukey's post hoc test used to correct for multiple comparisons and two‐way student's *t*‐test used in instances where only two groups are compared.

## 3 RESULTS

### 3.1 Valsartan attenuates MFS‐associated aortic root widening at sub‐BP‐lowering doses

**FIGURE 1:**
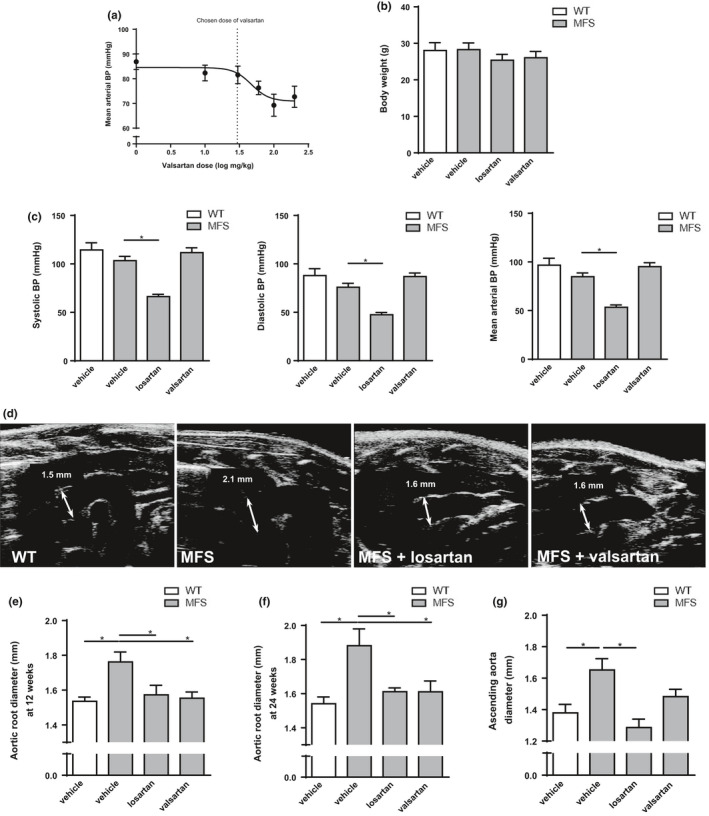
Valsartan attenuates aortic root widening in MFS mice independent of BP lowering. (a) Dose‐response curve for valsartan was performed to determine the highest possible non‐BP lowering dose. (b) Long‐term treatment with ARBs resulted in no change in body weight between groups. (c) Losartan treatment results in reduced systolic, diastolic, and mean arterial BP in MFS mice, whereas valsartan does not affect BP. (d) Representative echocardiograms of aortic roots of WT controls and MFS mice treated long‐term with losartan and valsartan. Treatment with both losartan and valsartan results in reduced aortic root diameter in MFS mice at (e) 12 and (f) 24 weeks of age. (g) Valsartan does not attenuate ascending aortic diameter compared to losartan in MFS mice at 24 weeks of age

A valsartan BP lowering dose‐response curve was established to determine the highest possible dose (30 mg/kg/day) with no significant effect on BP at 16 h post‐titration initiation (Figure 1a). This dose of valsartan was then compared to an established (Habashi, 2006; Sellers et al., 2018; Yang et al., 2009) dose of losartan (0.6 g/L) over a 18‐week treatment period. No changes in body weights were observed between treatment groups at 24 weeks of age (Figure 1b). MFS mice showed similar BP to WT mice, and treatment of MFS mice with losartan reduced systolic, diastolic, and mean arterial BP by approximately 48, 41, and 43 mmHg, respectively (Figure 1c), whereas valsartan at our pre‐determined dose (30 mg/kg/day) caused no significant changes in BP (Figure 1c). Echocardiography measurements revealed that untreated MFS mice showed the expected significant increase in aortic root diameter at 12 weeks (1.76 vs. 1.53 mm) and 24 weeks (1.88 vs. 1.54 mm) compared to WT controls (Figure 1d–f). In agreeance with previous observations (Habashi, 2006; Sellers et al., 2018), losartan treatment in our study significantly reduced MFS aortic root widening at 12 and 24 weeks by 83% and 79% back to untreated WT control levels, respectively (Figure 1d–f). Interestingly, treatment of MFS mice with valsartan at a dose that did not affect BP resulted in similar inhibition of aortic root widening as that observed with losartan (Figure 1d–f). Thus, these data show that valsartan is as effective as losartan at reducing MFS‐associated aortic root widening at a dose of valsartan that does not lower BP, thereby uncoupling BP‐lowering effects from ARB‐mediated inhibition of aortic root widening.

### 3.2 Valsartan ameliorates MFS aortic root histopathology and ERK signaling at sub‐BP‐lowering doses

**FIGURE 2:**
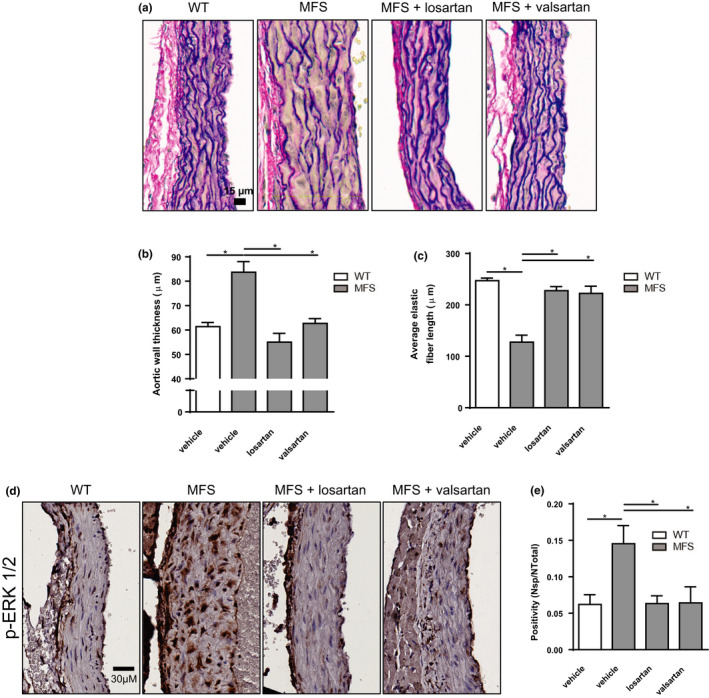
Valsartan attenuates MFS‐associated pathological remodeling and signaling in the aortic root of MFS mice. (a) Representative Van Geison's staining of aortic roots of WT and MFS mice treated long‐term with losartan or valsartan. Treatment with both ARBs (b) decreases aortic wall thickness and (c) increases average length of elastic fibers in the aortic root of MFS mice. (d) Representative p‐ERK ½ staining, and (e) average quantification of aortic roots of WT and MFS mice treated long‐term with both ARBs

Following euthanasia at 24 weeks of age, histological analyses of aortic sections revealed MFS‐associated medial thickening (83 vs. 61 μm) and average elastic fiber length (128 vs. 247 μm) compared to untreated WT mice (Figure 2a–c). Treatment with a BP‐lowering dose of losartan fully prevented MFS‐associated medial thickening and reduced average elastic fiber length relative to untreated WT levels (Figure 2b). Despite a lack of BP lowering effects, treatment with low‐dose valsartan reduced aortic root thickness and improved elastic fiber fragmentation similarly to losartan (Figure 2a–c).

To assess the sub‐BP lowering effect of valsartan on pathological aortic signaling, non‐canonical TGF‐β signaling was assessed though mitogen activated protein kinase ERK1/2 phosphorylation quantification. Sections obtained from losartan and valsartan‐treated groups revealed that both treatments completely **reversed** the significant increase of ERK phosphorylation observed in MFS aortic roots at 24 weeks of age (Figure 2d and e). Hence, a sub‐BP dose of valsartan prevents MFS‐associated non‐canonical TGF‐β signaling in the aorta. Taken together with the echocardiography data (Figure 1), this supports that a sub‐BP‐lowering dose of valsartan is capable of preventing MFS medial remodeling and aortic root disease.

### 3.3 Sub‐BP‐lowering dose of valsartan activates NO‐dependent endothelial function

**FIGURE 3:**
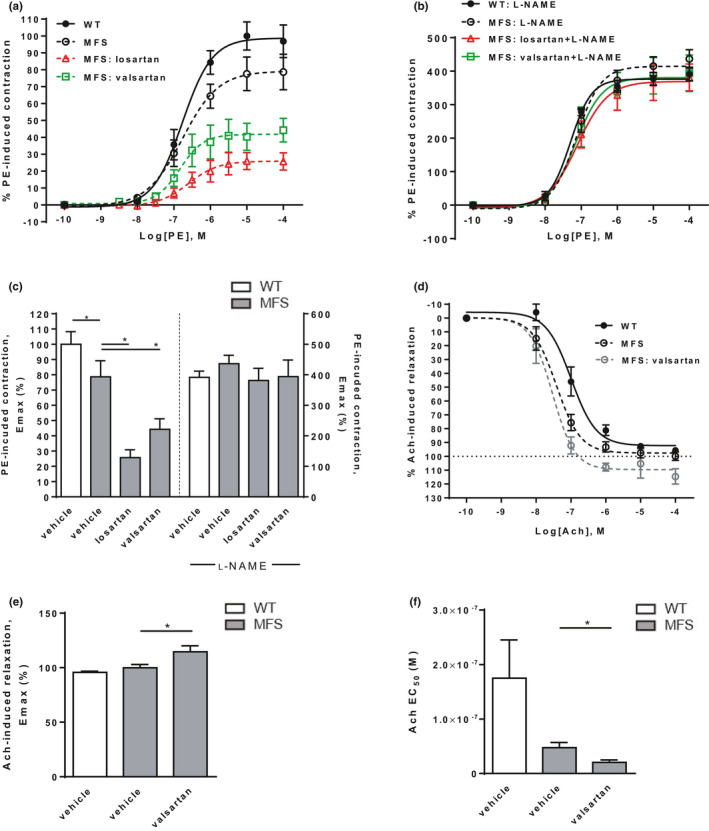
Losartan and valsartan treatment leads to enhanced endothelial function in the aorta of MFS mice. (a) Long‐term administration with losartan and valsartan reduces PE‐induced contraction of MFS mouse aorta. (b) Pre‐treatment with the NOS inhibitor L‐NAME increases PE‐induced in the controls and abolishes the ARB‐induced decrease in force development. (c) *E*_max_ values in the absence and presence of L‐NAME of WT and MFS aorta from vehicle and ARB treated mice. (d) Long‐term administration of valsartan increases Ach‐induced relaxation of MFS mouse aorta. Ach‐induced relaxation (e) *E*_max_ and (f) EC_50_ values of MFS mice aorta treated with valsartan

**FIGURE 4:**
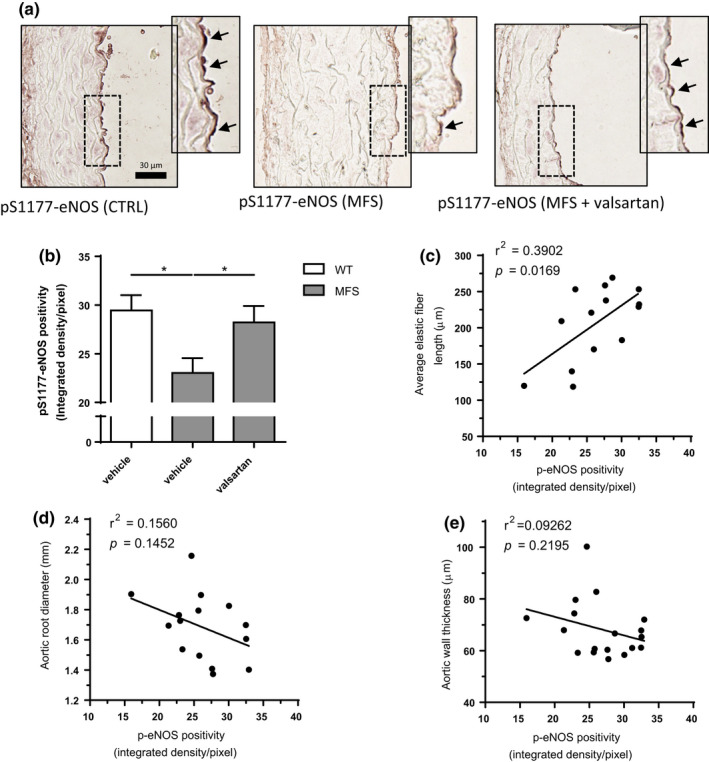
Valsartan treatment rescues phospho‐eNOS expression in the aorta of MFS mice. (a) Representative p‐eNOS staining, and (b) average quantification of aortic roots of WT and MFS mice treated long‐term with valsartan. p‐eNOS expression is plotted against a number of different aortic parameters assessed in this study to determine possible correlations between the two. From the aortic parameters that were compared to p‐eNOS expression, (c) average elastic fiber length was found to be significantly correlated whereas (d) aortic root diameter and (e) aortic wall thickness was not significantly associated with p‐eNOS levels

Current data support that enhanced endothelial function can be protective against MFS‐associated aortic dilation, an effect previously demonstrated with losartan (Sellers et al., 2018). As such, we used small chamber myography experiments to compare whether the sub‐BP lowering dose of valsartan can activate NO‐dependent endothelial function to levels comparable to that of losartan. The pharmacological response of aortic rings from WT mice was used as a baseline measurement. Compared to 24 week‐old WT aortic rings, MFS rings showed lower contractility to PE (10^−10^–10^−4^ M) (Figure 3a, white circles vs. black circles), a typical feature of MFS‐associated vascular dysfunction, which was fully reversed by the non‐specific NOS blocker L‐NAME (10^−4^ M; Figure 3b). Interestingly, aortic rings from MFS mice treated with losartan or valsartan demonstrated much lower contractility in response to PE (Figure 3a) in a fully L‐NAME reversible fashion (Figure 3b), indicating NO‐dependent endothelial function activation. Compared to the maximum PE‐induced constriction in untreated WT tissues (*E*_max_), untreated MFS tissues showed a 22% decrease in *E*_max_ whereas aortic rings from MFS mice treated with losartan and valsartan demonstrated significant 74% and 66% reductions in PE *E*_max_ (Figure 3c, left axis) and this was fully normalized with L‐NAME (right axis). Furthermore, Ach‐induced endothelium‐dependent relaxation was significantly improved in MFS mouse aortas after long‐term treatment with valsartan (Figure 3d–f). Unfortunately, technical limitations precluded Ach response measurements in the losartan treated setting. Hence, valsartan, even at non‐BP lowering doses, prevents PE‐induced constriction in a NO‐sensitive manner and potentiates Ach‐induced vasodilation, confirming endothelial function activation independent of valsartan's BP‐lowering effects. Given the NO‐dependent reduction in vessel contractility with a sub‐BP lowering dose of valsartan, we assessed the phosphorylation levels of eNOS, the primary source of NO in blood vessels. Sections obtained from valsartan‐treated groups revealed that valsartan reversed the significant decrease in eNOS phosphorylation observed in MFS aortic roots at 24 weeks of age (Figure 4a and b). Increased levels of p‐eNOS in the aortic tissue showed a positive correlation with increased elastic fiber length (Figure 4c), an indicator of reduced elastic fiber fragmentation, but not reduced aortic root widening or aortic wall (Figure 4d and e).

## 4 DISCUSSION

Despite early optimism, losartan's underwhelming efficacy at preventing MFS aortic root remodeling in larger clinical studies (Habashi, 2006) has helped create a shroud of ambiguity over its use and how to improve patient outcomes (Forteza et al., 2016; Lacro et al., 2014). In a recent study, we provided evidence that afterload reduction through BP‐lowering may be of low therapeutic value in MFS and that losartan‐mediated aortic root stability may be the result of protective ATR1‐independent endothelial function activation (Sellers et al., 2018). These findings would be in line with previous work demonstrating that loss of endothelial flow‐mediated dilation correlates closely with aortic dilation in MFS patients (Takata et al., 2014). Furthermore, compounds known to activate endothelial cell function and NO have shown promising results in MFS mice, lending credence to our previous findings (Hibender et al., 2016; Wallerath et al., 2002). However, simple activation of NO‐dependent vasodilatory signaling in SMC may be insufficient to promote aortic stability, as individuals with a protein kinase G1 activating mutation develop thoracic aneurysms and dissections (TAAD)(Schwaerzer et al., 2019) and show greater aortic oxidative stress, which is typically associated with reduced NO bioavailability. To our knowledge, the current study is the first to document a high degree of aortic root stability in MFS with the ARB valsartan along with evidence of chronic eNOS activation despite absence of BP lowering while reaching similar therapeutic efficacy to a more significant dose of losartan. These unexpected findings illustrate how fragmental our knowledge is of the process by which aortic root remodeling occurs in MFS, and suggest that ARBs may mediate their biological effects through more complex and heterogeneous means than anticipated.

From a clinical perspective, the concept of effective ARB‐mediated attenuation of MFS aortic root growth in the absence of BP lowering may represent a novel but unexpected opportunity to improve patient management. Recent MFS management trials have helped cast doubts about the causal role of BP in aortic root widening, where studies have found no correlation between changes in BP‐lowering and changes in aortic root diameters in patients treated with losartan versus atenolol (Andel et al., 2020; Forteza et al., 2016; Groenink et al., 2013). These doubts have been further materialized in pre‐clinical settings using rodent models of MFS. For example, calcium channel blockers have been shown to worsen aortic root widening in MFS mice despite lowering BP to a similar degree as losartan (Doyle et al., 2015). Additionally, exercise can attenuate rodent MFS aortic root widening despite an increase in systolic BP (Gibson et al., 2017; Mas‐Stachurska et al., 2017). There is also evidence to suggest that a non‐BP‐lowering dose of valsartan was sufficient to inhibit rat aortic abdominal aneurysm (Fujiwara et al., 2008). Since endothelial function activation, rather than BP lowering, may be required for losartan to inhibit MFS aortic widening, the poor efficacy of losartan in MFS clinical trials may be due to difficulty of reaching an endothelial function‐enhancing dose, whereas valsartan may have a greater effect on endothelial function than losartan. Using an established method of allometric dose scaling (Nair & Jacob, 2016), the commonly used rodent dose of losartan reported by most studies is equivalent to roughly 7 times that of current recommended maximum dose of 100 mg/day losartan in the clinic (Habashi, 2006; Sellers et al., 2018; Yang et al., 2009). In contrast, the dose of valsartan (30 mg/kg/day) used in this study translates to a human equivalent dose within the acceptable maximum recommended dose (320 mg/day). Hence, valsartan could be a better drug candidate for future human MFS clinical outcomes or quality of life studies. Chronic use of losartan has non‐serious but non‐negligible side effects that are, in part, related to its reduction of afterload, such as tiredness and lack of energy, which affects quality of life and patient compliance (Ratiu et al., 2018). If explored in MFS patients, the current work on sub‐BP‐lowering doses of valsartan may result in improved outcomes without BP‐related side effects and potentially lead to reconsideration of current guidelines.

The present myography and eNOS phosphorylation assessment studies also suggest that ARBs have more complex pharmacodynamic properties than simply reducing BP. It remains to be determined whether the relatively high degree of endothelial function activation induced by valsartan can participate to its overall BP‐lowering effect when used at higher doses. While NO released from aortic rings is a vasodilatory mediator, control of BP tends to occur primarily in resistance arteries rather than larger conduit vessels like the aorta. On the other hand, NO activation by valsartan could also trigger compensatory vasoconstriction, resulting in no net change in BP. How the modulation of BP and endothelial function in mice translates to humans is poorly understood, especially with ARBs. A potential point of contention is the heightened release of reactive oxygen species by a dysfunctional endothelium, events implicated in both MFS and non‐MFS thoracic aortic aneurysms (Chung et al., 2007; Ejiri et al., 2003; Jiménez‐Altayó et al., 2018; Syyong et al., 2009; Wilson et al., 1999) as well as heterogeneities in the way the aortic root—the main site of aortic complications in MFS—and the ascending aorta react to NO synthesis inhibition in MFS mice (Sellers et al., 2018; Oller et al., 2017). These two sections of the aorta different in their embryonic origins and may therefore behave differently, and this may hold true for the descending aorta as well, which highlights a limitation of our approach that correlates descending aorta myography results with aortic root measurements. Nonetheless, it must be noted that increasing eNOS‐derived NO release in a redox‐independent fashion in the descending aorta ex vivo as well as in vivo using experimental eNOS‐activating peptides (Bernatchez et al., 2011) correlated well with inhibition of MFS aortic root widening (Sellers et al., 2018). Although both losartan and valsartan activate vasodilatory NO, they differ slightly structurally; both are part of the tetrazole‐containing ARBs but losartan contains an imidazole moiety and is synthesized as a salt whereas valsartan has a more lipophilic structure including a carboxylic acid chain that in fact resembles the active metabolite of losartan (EXP3174). ARB metabolism is complex and, while little is known of valsartan metabolites, losartan has many active metabolites that differ in their intracellular and extracellular signaling properties (Kappert et al., 2009). How these metabolites can influence aortic remodeling under normal and blunted FBN1 expression is unclear, and whether other ARBs possess active metabolites that could regulate aortic wall remodeling remains to be determined.

## CONFLICT OF INTEREST

The Authors declare that no conflicts of interest exist.

## AUTHOR CONTRIBUTION

AYT, ZW, MAS were responsible for data acquisition and analyses. PB, ZW and ME were responsible for funding. All authors reviewed and edited the final manuscript.
